# *Mycobacterium avium* subspecies *paratuberculosis* infection in cattle – a review in the context of seasonal pasture-based dairy herds

**DOI:** 10.1186/s13620-022-00217-6

**Published:** 2022-05-20

**Authors:** Niamh L. Field, Conor G. McAloon, Lawrence Gavey, John F. Mee

**Affiliations:** 1 Animal and Bioscience Research Department, Teagasc, Moorepark Research Centre, Fermoy, Co. Cork P61 P302 Ireland; 2 UCD School of Veterinary Medicine, University College Dublin, Belfield, Dublin, D04 W6F6 Ireland; 3 Animal Health Ireland, Carrick-On-Shannon, Ireland

**Keywords:** Paratuberculosis, Pasture-based, Seasonal, Review

## Abstract

Johne’s disease is an infectious disease affecting cattle, other ruminants and non-ruminant wildlife worldwide, caused by *Mycobacterium avium* subspecies *paratuberculosis* (MAP). This review provides an up-to-date concise overview of the pathogenesis of MAP, the significance of Johne’s disease in cattle and the use of diagnostic testing at both animal and herd level in the context of seasonal pasture-based herds. While MAP can only replicate intracellularly, the bacterium is sufficiently robust to survive for months in the environment. Transmission of MAP is mostly via the faecal-oral route, however in-utero transmission in also possible. The bacteria evade the immune system by persisting in macrophages in the small intestine submucosa, with this latent stage of infection lasting, in most cases, for at least two years before bacterial shedding and clinical signs begin. The slowly progressive nature of MAP infection, poor performance of diagnostic tests and management systems that expose susceptible calves to infection make control of Johne’s disease challenging, particularly in seasonal calving herds. Testing of individual animals provides little assurance for farmers and vets due to the poor sensitivity and, in the case of ELISA, imperfect specificity of the available tests. Repeated herd-level testing is utilised by the IJCP to detect infected herds, identify high risk animals, and provide increasing confidence that test-negative herds are free of infection. The IJCP aims to control the spread of Johne’s disease in cattle in Ireland, in order to protect non-infected herds, limit the economic and animal health impact of the disease, improve calf health and reassure markets of Johne’s disease control in Ireland.

## Introduction

Johne’s disease caused by *Mycobacterium avium* subspecies *paratuberculosis* (MAP) affects various domesticated and wild species worldwide. MAP is an obligate intracellular pathogen incapable of environmental replication [42]. Numerous strains of MAP affect many species including cattle, sheep, goats, deer and camels. Two distinct groups of strains have been identified using various molecular techniques but most recently using whole genome sequencing [80]. Type C or cattle-type strains are the main strains isolated from cattle, and Type S or sheep-type strains are the predominant strains isolated from sheep [11]. Commercially available culture and PCR tests cannot differentiate between strains. These strain types were initially thought to be host-adapted to either cattle or sheep. However further analysis of isolates from various species around the world has shown that Type C strains in particular show no host preference, and are capable of inter-species transmission [80]. Type S strains, while mostly isolated from sheep and goats, have been reported to have infected beef cattle in New Zealand and it is suggested interspecies transmission of S-type strains is possible where there is close contact between species [87].

While MAP cannot proliferate on its own outside a host, the organism is sufficiently robust to survive in the environment for prolonged periods. In Australia, two studies demonstrated MAP could survive for 13 weeks [20] and 16 weeks [95] in 70% shade, and an S-strain survived for 55 weeks in 100% shade [95]. It can therefore be concluded that environmental MAP can remain viable for many months and constitute a risk for transmission of infection to susceptible animals. The organism has been shown to remain mostly in the upper levels of soil and on grass, after application of artificially contaminated slurry, rather than moving down through the soil profile, even under rainfall conditions of 1000 mm/year [69]. This represents a potential hazard to susceptible animals grazing pasture recently treated with contaminated slurry. Grewal et al. [30] showed MAP could survive a minimum of eight weeks in simulated lagoon slurry samples, however there are no quantitative data on the survival time of MAP on surface pasture after spreading of contaminated slurry.

MAP is of particular significance in dairy herds due to impacts on animal health, associated production losses [24], and controversial associations with Crohn’s disease [89]. Control of Johne’s disease relies on a combination of herd testing to identify infection and biosecurity measures to reduce the transmission of disease within herds and between herds [26]. It is important for control to identify infected animals through testing before they become infectious, however the poor sensitivity of diagnostic tests for MAP makes this very challenging.

The prevalence of Johne’s disease in dairy herds in Ireland was estimated in 2005 (0.20) [27], and in 2016 (0.28) [50, 53]. Herd prevalence may have increased in recent years due to the significant herd expansion after milk quotas were abolished in 2015. Control of Johne’s disease is a priority for the Irish dairy industry [58]. As a pasture-based system, Ireland has an interest in capitalising on its environmental sustainability credentials in trade negotiations and the development of the Irish Johne’s Control Programme (IJCP) helps to support this.

Much of the published Johne’s disease research originates from countries with predominantly indoor housing, year-round calving systems. In this narrative review we will focus on Johne’s disease transmission, pathogenesis, diagnosis and control in the context of seasonal pasture-based dairy systems. For the purpose of the review, we have defined “pasture-based” as herds where cows have access to housing over the winter, as is most commonly seen in Irish dairy herds.

## Transmission

MAP bacteria are primarily transmitted orally in faeces, colostrum or milk from an infectious animal. The ileum and jejunum are the main portals of entry. The organism crosses the epithelium via peyers patches and is then scavenged by macrophages where it can survive and evade the immune system [81, 82].

MAP is primarily shed in the faeces of infected animals, with the majority of infectious animals in a herd being classified as low and intermittent shedders [15, 56, 93]. Faecal shedding from adult cows is the primary route of transmission to calves, with much of the emphasis for control of MAP being put on hygiene measures at calving and in the calf environment. MAP is also shed in milk and colostrum from infected cows. In a study by Stabel et al. [78], MAP was detected by direct PCR in 49.2 and 12.6% of milk samples collected from cows in the clinical (shedding > 100 cfu/g faeces and symptomatic) and subclinical (shedding < 10 cfu/g faeces and asymptomatic) stages of JD, respectively, demonstrating the potential for calves to become infected by this route. In the same study, MAP shedding in colostrum from clinical and subclinical cows was quantified at 250 cfu/ml and 24 cfu/ml, respectively.

Farmers are advised not to pool colostrum fed to calves and to feed milk replacer instead of whole milk to reduce the risk of transmission by this route. There are particular challenges for seasonal herds posed by the high risk of MAP transmission during calving and early calf feeding. With compact calving in the spring, particularly if all cows calve indoors, it is challenging for farm management to maintain high standards of hygiene. It is also, in many cases, impractical to avoid pooling of colostrum. However, a recent review of the epidemiology of MAP [52] indicated that direct shedding of MAP into colostrum and milk is of lesser importance than faecal shedding as a transmission route. The authors held the view that the message to farmers and their service providers should be that MAP is primarily transmitted faecal-orally.

Another documented route of infection is via in-utero transmission. A meta-analysis of the literature [96] estimated that 9% and 39% of calves from subclinically infected and clinically affected cows, respectively, are born infected. The estimates were found to vary depending on the within-herd prevalence of infection and the ratio of subclinical to clinical infections. The mechanism of infection by this route is unknown.

## Pathogenesis

The progression of MAP infection can be described according to target conditions for diagnostic testing as described by Nielsen and Toft [62]. This categorises the infection into three stages: infected, infectious and affected. There is some overlap between these target conditions with affected animals also being both infectious and infected, and infectious animals also being infected. This categorisation of the stages of MAP infection is useful when evaluating diagnostic tests, and will be used here to describe the pathogenesis of the infection.

### Infected

Once MAP reaches the epithelium of the ileum it is taken up by M cells located in Peyer’s patches and is then engulfed by macrophages [57, 99]. The bacteria are capable of persisting within the macrophages in the submucosa and evading the animal’s immune response, thus leading to latent infection in the small intestine. The mechanisms by which MAP can remain hidden within macrophages include: inhibition of phagosome acidification and phagolysosome fusion, enhancing macrophage secretion of IL-10 and blocking the ability of infected macrophages to be activated by gamma interferon (IFN-y) for the clearance of the intracellular pathogen [2].

The presence of MAP in the submucosa attracts more macrophages, giant cells and lymphocytes to the area, resulting in granulomatous inflammation. While this inflammation serves to initially contain the infection, it also disrupts the structure of the surrounding tissue, thereby affecting function [81]. By Nielsen and Toft’s [62] definition, animals in the infected stage do not shed any MAP, and once shedding commences are then defined as infectious.

### Infectious

As the infection progresses the cell-mediated response wanes, allowing further dissemination of the organism in the tissues of the animal, and signalling the beginning of faecal shedding and the onset of antibody production [81]. This switch in immune response has also been associated with a shift from subclinical to clinical stages [77], although the timing of progression in immune responses and disease stage is not fully understood. A longitudinal study found that the age at which antibodies were first detectable in milk from naturally-infected cows ranged from 2–11 years old, with the highest probability of testing antibody positive between 2.5–4.5 years old [59]. Studies where faecal shedding levels of MAP from animals were quantified have shown that the majority of animals shedding MAP in a herd are low-shedders (< 10 cfu/tube) with a smaller proportion designated as high-shedders (> 50 cfu/tube) [15, 93]. A study by Mitchell et al. [56] also determined that an intermittent pattern of shedding was more common in the study population than continuous shedding. Schukken et al. [71] further described these intermittent shedders as “non-progressors”, where there was no increase in the amount of MAP shed over time, and there was an absence of shedding in between samples that showed MAP shedding. In these non-progressors there was a virtual absence of a detectable humoral immune response. Similarly, Nielsen [60] demonstrated during a longitudinal study that antibodies to MAP can be detectable by ELISA prior to shedding of MAP, but the probability of detecting antibodies increases with the progression of shedding from transient and intermittent to continuous and high shedding.

### Affected

The length of time between the start of faecal shedding of MAP and the onset of clinical signs is variable. Some animals will develop overt disease within six months of becoming infectious, while others may shed for several years without displaying clinical signs [81, 92]. Progressive granulomatous inflammation in the small intestine causes malabsorption with resultant diarrhoea (cattle only), weight loss and protein-losing enteropathy [81]. Gross lesions in the majority of animals are confined to the distal small intestine, not extending past the ileocaecal valve, i.e. the ileum, and mesenteric lymph nodes [8, 92] with thickening, corrugation and reddening of the intestinal mucosa and lymph nodes becoming swollen and pallid. Histopathology of affected tissues reveals granulomatous inflammation involving giant cells and macrophages in the lamina propria of intestinal villi and the submucosa [8]. A recent study demonstrated that disease state (subclinical vs clinical disease) may be predicted based on numbers of Th1-type cells and macrophages at the site of infection, with further investigation required to determine if a diagnostic assay could be developed to measure these markers ante-mortem [36].

## Diagnostic testing

Diagnostic tests for MAP can be divided into direct methods that detect the organism, and indirect methods that detect the immune response to the organism. There is no gold standard reference test for MAP as all test methods typically have low sensitivity (Se) and/or imperfect specificity (Sp) in individual animals. ELISA testing, faecal culture or PCR and pathology are most commonly reported as diagnostic tests for MAP worldwide, while agar gel immunodiffusion, faecal Ziehl–Neelsen smear, complement fixation testing and intradermal skin testing are least commonly used [94]. This review will focus mainly on tests that are commercially available and approved for use in Ireland, while briefly summarising novel techniques.

### Direct test methods

(i) Post-mortem pathology and tissue culture
  The nearest thing to a reference standard method of diagnosing MAP in an individual animal is on post-mortem investigation. The ileum, jejunum, ileo-caecal valve and mesenteric lymph nodes are the tissues most commonly culture-positive for MAP [82]. A combination of observation of gross lesions such as thickening of the intestine, reddening and corrugation of the intestinal lining, laboratory testing of target tissues by histology, culture and/or PCR is used to make the diagnosis. While it is the reference standard diagnostic method for MAP infection, post-mortem testing, either on-farm or in slaughterhouses, is not routinely practical as part of a testing strategy in a herd or national control programme.
(ii) Faecal culture
  Culture of faecal samples provides an option for diagnosis of MAP that is non-lethal and highly specific. There are various methods used to culture MAP including both solid media such as Herrold’s egg yolk medium and liquid media such as M7H9C. Collins et al. [12] and Alinovi et al. [1] estimated the Se of faecal culture to be 60% and 72% respectively. When broken down by target condition (infected, infectious, affected) by Nielsen and Toft [62], the Se varies, with infected animals having an estimated Se of between 23–29%. Norton et al., (2010) evaluated the effect of lactation stage on the Se of faecal culture in seasonal pasture-based dairy herds in New Zealand. The authors found a 32% increase in Se in early lactation (Se = 51.7%) compared to late lactation (Se = 19.8%), which may be attributable to increased stress associated with calving and peak milk production. The Sp of faecal culture for MAP is frequently assumed to be 100% as it relies on direct identification of the organism (as long as molecular methods such as PCR are used to confirm the isolates from culture are MAP). However, the Sp may be imperfect in infected populations due to the phenomenon of pass-through whereby non-infected animals in an environment contaminated with MAP theoretically may ingest the bacteria, which could subsequently be passively shed into faeces [83].
(iii) Faecal PCR
  The accuracy of PCR tests for MAP appears to have improved greatly in recent years. Collins et al. [12] produced consensus recommendations for testing cattle for MAP in the USA that reported the estimated Se of PCR testing to be 30%, with faecal culture significantly higher at 60%. Alinovi et al. [1] determined that the test Se of a real-time PCR kit was the same as solid culture at 72%. Two recent studies reveal improved Se estimates for commercially-available PCR kits. An evaluation study conducted by Prendergast et al. [66] compared the performance of three different real-time PCR kits against faecal culture. The Se estimates of the kits ranged from 73.5% to 93% in relation to culture, with Sp estimated at 99–100%. Similar results were obtained by Schwalm et al. [73], with two different PCR kits detecting 86% and 89% of MAP-positive faecal samples. A critical factor affecting the Se of PCR tests is the efficacy of the DNA extraction process. PCR inhibitors present in faeces and the characteristics of the cell wall of MAP can lead to difficulty in extracting the DNA. Leite et al. [47] compared the Se of six different extraction kits followed by analysis with PCR using naturally infected faecal samples. Se estimates for the various kits ranged from 17–94%. A study by Fock-Chow-Tho et al. [22] demonstrated similar variability with three different extraction kits having estimated Se values of 43%, 88% and 22%. Magnetic separation techniques such as peptide-mediated magnetic separation (PMS), applied to samples before PCR can help to increase the Se of the test [70, 88], although to the authors’ knowledge there are no commercially available tests for MAP that utilise PMS currently.
  The reported estimates for the Se of faecal PCR for MAP are summarised in Table 1.

**Table 1:** Reported estimates for the sensitivity of faecal PCR testing for MAP in relation to faecal culture

Estimated sensitivity of faecal PCR (%)	Source
73.6^a^	(Schwalm et al., 2019) [72]
86 – 89	(Schwalm et al., 2018) [73]
73.5 – 93	(Prendergast et al., 2018) [66]
22 – 88	(Fock-Chow-Tho et al., 2017) [22]
17.6 – 94.1	(Leite et al., 2013) [47]
72	(Alinovi et al., 2009) [1]

^a^liquid faecal culture used as reference standard
(iv) Phage-based assays

The use of bacteriophage assays to detect viable MAP is a relatively new technique that is currently being evaluated for use as a novel diagnostic test. Various assays have been evaluated and all are based around the D29 mycobacteriophage, which targets a range of mycobacteria species. In order to make the assay specific to MAP, an additional PCR step is applied in most published methods [28]. A recent study conducted in Northern Ireland evaluated a peptide-mediated magnetic separation (PMS) technique prior to using culture and a phage-based assay for MAP to test milk samples from dairy herds [63]. The estimated sensitivities of the PMS-culture and PMS-phage assay were 25% and 32.5%, respectively. The estimated Sp of the phage assay was 100%, while the PMS-culture was estimated to have 96% Sp. Further evaluation and validation of the phage-based assays on various sample matrices (milk, faeces) is required to determine their suitability as a new diagnostic test for MAP.

### Indirect test methods

(i) ELISA
  The most commonly used indirect test method for MAP is the ELISA, performed on either serum or milk samples. For the target condition “infected” the estimated Se and Sp of serum ELISA testing is 7–22% and 85–100% respectively [62]. There is a significant association between stage of lactation and antibody response, with typically higher antibody concentrations in early lactation and again in late lactation [51]. This may be more significant in seasonal systems where all animals are generally in the same stage of lactation at the time of sampling for a whole-herd test. ELISA testing for MAP using milk samples is a cost-effective and convenient option for dairy farmers who are already conducting regular milk recording (for volume, fat and protein content and somatic cell count) of their herds. In the Irish Johne’s Control Programme 47% of ELISA tests (*n* = 105,642) conducted in the 2020 programme year were done using milk samples [25]. Table 2 summarises the previously reported test characteristics (Se and Sp) for detecting MAP-infected animals with milk ELISA testing. Se estimates relative to faecal culture typically range from 21–30%.

**Table 2:** Reported test characteristics for milk ELISA tests relative to faecal culture and serum ELISA

Sensitivity relative to FC (%)	Sensitivity relative to serum (%)	Specificity relative to FC (%)	Source
30	-	-	(Laurin et al., 2017) [43]
22–25	-	99.6	(Lavers et al., 2015) [45]
-	87	99.8	(van Weering et al., 2007) [86]
21.2	45.7	-	(Lombard et al., 2006) [48]
28.85	-	99.7	(Collins et al., 2005) [13]
61.1	41.5	-	(Hendrick et al., 2005) [32]
(ii) Gamma-interferon

The gamma-interferon test (IFN-ƴ) uses an ELISA to detect IFN-ƴ in blood. There have been few publications evaluating the test in cattle [34, 37]. It can potentially detect MAP infection earlier than the ELISA antibody test as it detects the initial cellular immune response. The reported Se and Sp ranges from 13–85% and 88–95%, respectively [62]. Having lower Sp than the ELISA tests, it has been suggested that repeated testing to identify a rising titre may be appropriate when using this test to ascertain disease status [5].

## Herd-level test methods

A single negative test result from an individual animal provides little assurance against the risk of infection due to the low Se of diagnostic tests to detect MAP infection in individual animals, especially since testing for assurance or movement is typically conducted on young, pre-breeding animals for which test sensitivity is particularly low. Assurance and control programmes therefore are based on repeated testing and infection classification at herd-level [26]. The more negative herd test results a herd has, over time and depending on herd size, the greater the confidence one can have that the herd is free from infection. Modelling of various national surveillance strategies for the Irish dairy industry by Meyer et al. [55] demonstrated increased herd-level Se and reduced time to achieve 95% confidence of freedom in test-negative herds when conducting herd testing twice a year instead of once a year. Reducing the frequency of testing to every second year also significantly increased the time taken to achieve 95% confidence of freedom. The chosen method for testing a herd or a population of herds in a control programme will depend on the available resources as well as consideration of the test characteristics (Se and Sp). For seasonal calving systems, the timing of herd testing will be influenced by the need to avoid testing during busier times of year in the case of blood sampling, or the dry period in the case of milk sampling. Table 3 is adapted from a recent systematic review [21] summarising the published estimates for the sensitivity and specificity of herd-level tests for Johne’s disease (herd sensitivity (HSe) and herd specificity (HSp)). It is important to note that factors including herd prevalence, within-herd prevalence, choice of reference test and differing test protocols can affect Se and Sp estimates between studies.

**Table 3:** Reported test characteristics for herd-level tests for MAP, adapted from [21]

Screening test	Herd sensitivity (%)	Herd specificity (%)
Whole-herd ELISA^a^	56–95	0–96
Whole-herd ELISA + PCR^a^	60–86	100^c^
BMT ELISA	8–30	95–100
BMT PCR/culture	0–77	100^c^
Pooled faecal testing^a^	54–94	100^c^
Environmental sampling^b^	24–79	100^c^

^a^From field studies using culture methods as a reference test

^b^From studies evaluating a protocol using six composite samples

^c^Herd specificity can be assumed to be 100% due to direct detection of MAP bacteria

### Whole-herd individual serology

This is a commonly used herd testing strategy in many countries [94]. Individual blood or milk samples are obtained from all adult animals in a herd, and samples are tested by ELISA for antibodies to MAP. In order to determine if a herd is likely infected, a cut-off in seroprevalence may be defined. Previous studies have reported cut-offs of > 0% seroprevalence, > 2% seroprevalence or > 3% seroprevalence [44, 48, 91]. The HSe of this herd test method is relatively high, ranging from 56–95% (Table 3), and may vary according to herd prevalence and within-herd prevalence of infection. However, due to the relatively low Sp of individual serum/milk ELISA testing, the HSp is typically imperfect, e.g. 95%. HSp can be increased to 100% by the addition of confirmatory faecal culture/PCR of ELISA-positive animals [74, 91]. Control programmes in two seasonal pasture-based systems, Ireland and New Zealand, have utilised variations on whole-herd ELISA testing with confirmatory faecal PCR for positive animals as a herd testing strategy. This strategy is associated with a reduction in HSe, and requires additional expense and animal handling compared to ELISA alone.

### Bulk milk tank testing

Samples of milk from bulk milk tanks (BMT) can be tested by ELISA for antibodies to MAP, or directly using culture or PCR techniques. Studies investigating the HSe and HSp of this test often initially determine an optimal S/P (sample to positive) ratio using ROC analysis [61, 86]. The manufacturer recommended cut-off point is usually 30% S/P. At this S/P ratio, HSe estimates for BMT ELISA range from 8–30%, and HSp ranges from 95–100% [21]While having very low sensitivity, BMT ELISA is an inexpensive test requiring minimal effort to perform on a large scale. It may be a useful tool to identify high-risk herds for recruitment into control programmes, although care must be taken in interpreting results from herds engaging in tuberculin skin testing for *Mycobacterium bovis* due to the impact on specificity of the MAP ELISA after the skin test.

Some studies have evaluated the use of PCR and culture for identifying MAP in bulk milk tank samples. Stabel et al. [79] reported zero positive BMT culture tests from 37 infected herds, while Foddai and Grant [23] reported 50% detection rate in BMT samples using a liquid culture with peptide-mediated magnetic separation. Estimates for HSe of BMT PCR are also mixed ranging between 13–77% [14, 35, 79]. The potential for milk filter socks to be tested by culture or PCR has also been evaluated in two relatively small studies with HSe estimates of 61% for culture [9] and 85% for PCR [76].

### Pooled faecal testing (PFT)

Among the herd test methods with 100% HSp, testing pooled faecal samples for MAP by culture or PCR has the highest HSe of any test method other than individual faecal testing of all adult animals [74, 84]. HSp is assumed to be 100% and HSe ranges between 54–94% (Table 3). Various pool sizes have been evaluated but pools of five or 10 individual faecal samples are most commonly reported [14, 38, 54, 90]. Most studies used MAP culture methods rather than PCR for testing pooled faecal samples. The sensitivity of PCR relative to culture on pooled faecal samples was low in one study at 66% [39], although this is likely due to the use of a f57 PCR which is highly specific for MAP but less sensitive than IS900 PCR. PFT was determined to be a less practical and relatively expensive herd test method using modelling based on data from Irish dairy herds [74].

### Environmental sampling

Control programmes in USA, Canada, Australia, New Zealand, France and Germany use environmental sampling for the detection of infected herds [94]. This involves taking multiple samples of composite manure and slurry from areas of adult cow concentration (main cow housing, collecting yards) and manure concentration (slurry storage, slurry spreaders) and testing the samples by PCR or culture for MAP. Various numbers of composite samples have been evaluated but sets of six samples per farm is the most common protocol [46, 84, 97]. The reported HSe for six composite environmental samples is 24–79% and HSp is assumed to be 100% (Table 3. Environmental sampling requires further evaluation in seasonal pasture-based systems due to the potential impact this system may have on environmental contamination with MAP compared to indoor systems. Each sampling location will only have fresh manure available for certain months of the year (collecting yard in spring summer and autumn; calving pens in spring; cubicle housing in winter An alternative method of conducting environmental sampling is the use of boot swabs. Boot swabs are worn over boots while walking through areas of high cow and manure concentration and then removed and sent to the laboratory for testing by culture or PCR [19]. Three studies have shown them to be effective at detecting MAP in infected herds [18, 19, 98]. The majority of published studies evaluating environmental sampling originate in countries with indoor housing systems. A study is currently underway to evaluate this test in seasonal pasture-based herds in Ireland, with results expected to be published in 2022.

## The importance of national control of MAP

### Animal health and welfare

The direct effects of Johne’s disease on individual animals’ health and welfare are clear. It is believed that MAP infection also compromises the immune system of affected animals, leading to increased susceptibility to other infections. It has been shown in a number of longitudinal studies that there is an association between cows testing positive for MAP and increased somatic cell count [3, 49, 67]. In terms of seasonal pasture-based herds, Hoogendam et al. [33] found that MAP-positive herds in Ireland tended to have higher SCC than negative herds but the effect was not significant, and a more recent Irish study of 3,528 dairy cows [40] found no statistically significant association between Johne’s disease and SCC. However these were cross-sectional studies that would not have identified any associations that were separated in time. Therefore, while there is a probable association between the two conditions, there’s no evidence of a causal link between Johne’s disease and high SCC. Raizman et al. [68] also identified an association between clinical Johne’s disease and the incidence of pneumonia in animals on two large dairy farms.

### Economics

Johne’s disease causes a reduction in milk production [50, 53] and slaughter weight [41] in affected cattle, and premature culling leading to increased replacement costs [24]. Estimated economic losses due to Johne’s disease in published studies include $100 [64], $49 [85] and $44 [75] per cow per year in the USA, Canada and Australia, respectively. Data from pasture-based systems is limited. Kennedy et al. [40] identified no association between MAP ELISA status and milk production parameter in dairy cows (*n* = 3528) in Ireland. A study on a single large dairy herd in New Zealand found a significant association between subclinical MAP infection (test-positive on either ELISA or faecal PCR) and reduced milk production in individual cows [4]. A review of the literature by Garcia and Shalloo [24] concluded that the economic impact of Johne’s disease in herds with clinical cases is more pronounced than in herds with only subclinical infections. However as within-herd prevalence increases so does the cost. Donat et al. [17] demonstrated that there was a greater reduction in milk yield in faecal-culture positive cows as within-herd prevalence increased.

### Public health significance/perception

MAP was first associated with Crohn’s disease in humans in 1913 [16]. Several studies have tried to prove or disprove the theory that MAP infection in humans can cause the development of Crohn’s but to date there is no definitive evidence to back up this theory [10, 31, 65]. A recent meta-analysis [89] concluded that the zoonotic potential of MAP cannot be ignored, yet there was not enough evidence to show it has any impact on public health. MAP has been shown to occasionally survive the pasteurisation process, making milk from infected cows a potential hazard [29]. Viable MAP cells have also been detected in powdered infant formula that contains pasteurised milk products: 12.5% of powdered infant milk samples tested using a phage amplification assay combined with PCR contained viable MAP [7]. This is particularly concerning from an Irish perspective as exported specialised nutritional powders – or infant formula – were worth €929 million to the Irish economy in 2019 [6]. While further research is needed, MAP remains a potential zoonotic organism of significance for public health.

## The Irish Johne’s Control Programme

The estimated herd prevalence of Johne’s disease in the Irish dairy industry is approximately 30% [27, 50, 53]. This provides the Irish industry with a competitive advantage compared to neighbouring European and most other western nations having much higher prevalences. In 2009, industry experts and stakeholders in Ireland ranked Johne’s disease as an important disease requiring proactive management and mitigation [58]. The Irish Johne’s Control Programme (IJCP) was formed in 2013 as a collaboration between Irish industry stakeholders and the Department of Agriculture, Food and the Marine (DAFM) and is managed by Animal Health Ireland (AHI). It is available to dairy and beef cattle herds. In the context of the relative low herd prevalence of Johne’s disease in Ireland, the objectives of the IJCP are to 1) enhance the ability of participating farmers to keep their herds clear of Johne’s disease (JD), 2) assist participating farmers to reduce the level of infection in their herds, where present, 3) provide additional reassurance to the marketplace in relation to Ireland’s efforts to control Johne’s disease, 4) improve calf health and farm biosecurity in participating herds [25]. At the end of 2021 there were 1,987 dairy herds registered in the programme, representing approximately 13% of dairy herds in Ireland (L. Gavey, personal communication).

There are four programme requirements of all participating herds: 1) an annual whole-herd tests (WHT), 2) annual veterinary risk assessment and management plan (VRAMP), 3) ancillary faecal PCR test of any animal with an ELISA result of positive or inconclusive (unless the herd has previously had a positive result to an ancillary faecal test), and 4) a veterinary investigation (TASAH) of confirmed infection in a herd. An approved veterinary practitioner (AVP) who has received specific training conducts both the VRAMP and the TASAH investigation, and testing is undertaken by designated laboratories.

### Veterinary Risk Assessment and Management Plan (VRAMP)

Herdowners in the IJCP must participate in a detailed on-farm review and risk assessment conducted by their AVP. Management areas including bioexclusion, calf rearing, milk and colostrum feeding, management of weaned heifers, cow hygiene and calving practices are assessed by the AVP for risk of transmission of MAP. The outcome of the assessment is a set of farm-specific mitigation actions to reduce the risk of spread of MAP within the herd and/or risk of entry of MAP into the herd. The VRAMP is reviewed each year to monitor the herd’s progress and modify the management of risk. Most of the VRAMP is focussed on the high-risk period of calving when animals are fully housed and specific risks associated with pasture are also assessed: slurry spreading on pasture, importing of slurry and grazing of pasture with unknown MAP status.

### Herd testing

Herds registered in the IJCP must complete an annual whole-herd test (WHT), comprising MAP ELISA testing of all animals on the farm aged two years or more using either blood or milk samples. Any animal that has a positive or inconclusive ELISA result is required to be tested by PCR or culture for MAP bacteria with the aim of confirming infection in the herd. If any animals test positive on ancillary faecal testing for MAP, the herd is considered infected and no further faecal testing is required. For all herds, the IJCP advises that ELISA-positive or inconclusive animals that test negative on follow-up faecal testing should be considered “suspect” animals until the next herd test. Appropriate biocontainment measures such as removal or separation of high-risk cows, separate and clean calving areas, early removal after birth of calves from dams, hygienic collection and feeding of only low-risk colostrum and milk, and clean calf-rearing areas are advised for all herds and especially MAP-infected and suspect animals.

### Targeted Advisory Service on Animal Health (TASAH)

Following a positive faecal culture or PCR test, herdowners undertake a TASAH investigation funded by the Rural Development Programme. The TASAH investigation is conducted by a TASAH-trained AVP and involves an epidemiological assessment of the source, prevalence and spread of the infection, and a customised infection control plan to complement the VRAMP.

### Funding

The costs of these activities are supported through the programme. For all registered herds, the costs of required ancillary PCR testing and TASAH investigations are fully funded; for dairy herds only, the costs of VRAMPs are fully funded and milk processors provide assistance for the cost of the WHT.

### Programme results

Continued recruitment of herds into the IJCP is important for the credibility and sustainability of the programme but is also challenging, with new registrations fluctuating from 729 in 2019 to 139 in 2020 and 231 in 2021 (L. Gavey, personal communication). Out of 1760 herds registered in the IJCP in 2020, 1325 (75%) herds completed both the VRAMP and the WHT. In that programme year, 47% of ELISA testing was conducted on milk samples and 53% on blood samples, with 3.8% of ELISA tests overall being positive or inconclusive. Out of 5419 ancillary PCR tests in 2020, 281 (5.1%) were positive. Gavey et al. [25] observed that a higher proportion of milk samples test positive or inconclusive on ELISA each year compared with blood samples, and suggest that ELISA tests may have a slightly lower specificity on milk samples than for blood samples. However, the current push for increased uptake of milk recording in Irish dairy herds, to improve milk quality and to facilitate treatment of mastitis, presents an opportunity to attract more participants into the programme due to the relative convenience and lower cost of obtaining milk samples for ELISA compared to blood sampling.

## Conclusion

Much has been learned about the pathogenesis, diagnosis and control of Johne’s disease. The management practices of seasonal pasture-based dairy herds presents unique challenges and considerations for controlling MAP. Continued research in the context of seasonal pasture-based dairy herds will help to tailor control measures to these unique management systems, thereby ensuring the sustainability of control programmes into the future.

## Data Availability

Data sharing is not applicable to this article as no datasets were generated or analysed during the current study.
